# Changes in dietary habits after migration and consequences for health: a focus on South Asians in Europe

**DOI:** 10.3402/fnr.v56i0.18891

**Published:** 2012-11-06

**Authors:** Gerd Holmboe-Ottesen, Margareta Wandel

**Affiliations:** 1 Department of Community Health, Institute of Health and Society, University of Oslo, Oslo, Norway; 2 Department of Nutrition, Institute for Basic Medical Sciences, University of Oslo, Oslo, Norway

**Keywords:** dietary change, food habits, diabetes, cardiovascular disease, immigrants, South Asia, acculturation

## Abstract

**Background:**

Immigrants from low-income countries comprise an increasing proportion of the population in Europe. Higher prevalence of obesity and nutrition related diseases, such as type 2 diabetes (T2D) and cardiovascular disease (CVD) is found in some immigrant groups, especially in South Asians.

**Aim:**

To review dietary changes after migration and discuss the implication for health and prevention among immigrants from low-income countries to Europe, with a special focus on South Asians.

**Method:**

Systematic searches in PubMed were performed to identify relevant high quality review articles and primary research papers. The searches were limited to major immigrant groups in Europe, including those from South Asia (India, Pakistan, Bangladesh, Sri Lanka). Articles in English from 1990 and onwards from Europe were included. For health implications, recent review articles and studies of particular relevance to dietary changes among South Asian migrants in Europe were chosen.

**Results:**

Most studies report on dietary changes and health consequences in South Asians. The picture of dietary change is complex, depending on a variety of factors related to country of origin, urban/rural residence, socio-economic and cultural factors and situation in host country. However, the main dietary trend after migration is a substantial increase in energy and fat intake, a reduction in carbohydrates and a switch from whole grains and pulses to more refined sources of carbohydrates, resulting in a low intake of fiber. The data also indicate an increase in intake of meat and dairy foods. Some groups have also reduced their vegetable intake. The findings suggest that these dietary changes may all have contributed to higher risk of obesity, T2D and CVD.

**Implications for prevention:**

A first priority in prevention should be adoption of a low-energy density – high fiber diet, rich in whole grains and grain products, as well as fruits, vegetables and pulses. Furthermore, avoidance of energy dense and hyperprocessed foods is an important preventive measure.

Immigrants from low-income countries comprise an increasing proportion of the population in Europe. Over time, a mutual exchange of ideas and habits between immigrants and the host population has taken place. With regard to food habits, immigrants are influenced by the food culture of the majority, leading to changes in their dietary habits (1), while they contribute to widening the specter of new food items in shops and restaurants.

The issue of changes in dietary habits after migration has become pertinent, since it has been found that some ethnic minority groups are at higher risk than the host population in Europe of becoming obese and contract nutrition-related diseases, such as Type 2 Diabetes (T2D) and cardiovascular diseases (CVD) (2–5). Thus, immigrants may be in great need of nutritional advice and treatment. However, health personnel report that they often feel poorly equipped to cater to the needs of these groups in an adequate way due to insufficient knowledge of their diet and changes that have occurred after migration (6).

## Burden of obesity and diabetes in immigrants from low-income countries

The prevalence of obesity is increasing in both high- and low-income countries (7). In low-income countries, the prevalence is usually higher in urban areas, although the relative change is now higher in the rural areas in many countries and regions (8). Concomitantly, the prevalence of T2D and CVD is rising in low-income countries.

South Asians have been found to be susceptible to obesity (particularly central obesity), T2D, and CVD (9, 10). Genetic, epigenetic, and lifestyle factors, as well as gene–environment interactions, have been proposed to explain the high susceptibility to these diseases (11–13).

A few studies have compared Body Mass Index (BMI) and waist circumference between South Asians settled in Europe and their counterparts with similar cultural and genetic background in the country of origin: India (14), Pakistan (15), and Sri Lanka (16). These have consistently found higher BMI (2.1–5.8 BMI point difference) in the European Asians, compared to counterparts in their country of origin. Waist circumference was also found to be higher in the European Asians in most of these studies.

Regarding diabetes, a meta-analysis, including data from surveys in 199 countries, showed that there has been an increase in glycemia and in prevalence of diabetes in almost all regions of the world between 1980 and 2008, and that South Asia and the Middle East/North Africa are among the regions where the rise has been reported to be especially high (17). In 2011, the comparative (age adjusted) prevalence of diabetes was estimated to 9.2% in India, 8.0% in Pakistan, 8.1% in Turkey, and 11.3% in Iran (18).

In comparison, the age-adjusted prevalence of diabetes has been estimated to be around 20% among British South Asians, nearly fivefold higher than the indigenous population in Britain (12). In a study from Oslo, the age-standardized prevalence of diabetes differed significantly between five ethnic groups (women/men): Pakistan 26.4/20.0%, Sri Lanka 22.5/20.7%, Turkey 11.9/12%, Vietnam 8.1/10.4%, and Norway 2.7/6.4% (19). In a review on diabetes in the Nordic countries, the estimations of relative risk for South Asians compared to the native population were 5.6–7.5, and for those from the Middle East were 1.3–5.5 (20). The mortality rate ratios from diabetes were found to be especially high for migrants originating from the Caribbean and South Asia (21). This study, which included many immigrant groups in Europe, also found a consistent inverse association between Gross Domestic Product in the country of birth and diabetes mortality after migration. This finding suggests that socioeconomic change may be one of the key etiological factors.

Since prevalence of diabetes is increasing in many low-income countries, the difference between migrants and those who reside in the country of origin is not always large. In a study comparing Gujaratis in Great Britain (GB) with those in Indian Gujarat (IG), the prevalence of diabetes was high in both groups (women: 11.0% in IG and 16.6% in GB; men: 18.2% in IG and 19.6% in GB) (14). However, the prevalence of impaired glucose tolerance was lower among those residing in GB, though it was more frequently accompanied by excess metabolic CVD risk.

## Scope

It is well known that both diet and physical activity are important in the control of obesity and T2D (7). This article will focus on the diet, particularly on changes in dietary habits after migration, and the implications for nutrition and health among South Asians in Europe. First, the literature on the processes of change, and the dietary data describing the actual changes after migration, will be reviewed. Then, the evidence for a relationship between the dietary changes and the reported changes in disease patterns with a focus on T2D and its consequences for CVD will be discussed. Finally, the implications for preventive efforts will be suggested.

## Literature search

Systematic searches in PubMed were carried out to identify relevant systematic reviews and primary research papers, mostly population based observational studies using adequate data collection methods and analytical approaches. Papers suggesting theoretical models for understanding how and why dietary changes among immigrants take place were also included. The searches were focused on immigrants from South Asia (India, Pakistan, Bangladesh, and Sri Lanka). These were combined with search words such as diet, change, food, habits, nutrition, transition, acculturation, diabetes mellitus, metabolic syndrome (MetS), CVD, risk. The reference lists of the articles provided additional literature. Articles in English from 1990 and onwards from Europe were included. For health implications, mostly review articles and meta-analyses from 2007 and later, as well as studies of particular relevance to South Asians, were chosen.

## Processes of change in dietary habits

Two driving forces of change in lifestyle and health outcome regarding immigrants should be taken into account. One is the nutrition transition, which is a global trend. The other is the process of dietary acculturation which occurs with migration.

The nutrition transition is driven by industrialization and globalization of the food market. It started in high-income countries, but has spread to low-income countries in the last decades and has escalated, first in urban areas and also lately in rural areas (22). It has led to an increased supply of what has been described as ultra-processed foods, which are cheap, rich in fat, sugar, and other refined carbohydrates (8, 23). The result has been increased intake of energy-dense foods and snacks, sugary drinks, and a shift toward more meat and dairy. Concomitantly, the intake of whole grains and legumes is reduced (8, 23, 24). Urban areas may have high availability of fruits and vegetables, but prices determine access. Urbanization has led to less physical activity and thus a lesser need for food energy.

The other driving force – dietary acculturation – has been defined as ‘the process that occurs when members of a minority group adopt the food choices and eating patterns of the host country’ (1, 25). The concept ‘acculturation’ is multidimensional and includes changes in identity, attitudes, and values that accompany an individual's movement from their original culture toward the mainstream culture in the new country (26). Dietary acculturation does not appear to move linearly from one end of the acculturation continuum to the other. Available research indicates that immigrants may find new ways to compose traditional dishes and meals, to exclude foods, and consume new foods (27, 28).

Satia-Abouta et al. (1) have proposed a model of dietary acculturation which focuses on factors that may have an impact on the trajectories leading to new dietary habits. They suggest that socioeconomic and demographic factors together with the cultural factors that immigrants bring along influence the degree of exposure to the host culture, which, in turn, leads to changes in psychological factors, taste preferences, and changes in food procurement and preparation, and thus dietary changes.

Another model, developed by Koctürk-Runefors (29), focuses on the temporal process of dietary change and introduces ‘identity’ and ‘taste’ as two opposing forces affecting the change. Foods are divided into three main categories: ‘staple foods’ (carbohydrate-rich foods such as grains and tubers), ‘complementary foods’ (meat, fish, milk, eggs, vegetables, and lentils), and ‘accessory foods’ (fats, oils, herbs and spices, sweets, nuts, fruits, and drinks). It is further proposed that the staple foods are more closely tied to identity than the foods in the other categories, and will therefore be the last ones to change. Thus, the first change will be the intake of accessory foods, which are postulated to be less tied to cultural identity but driven by taste preference. Since these foods include fats, sweets and drinks, such changes may have large implications for health.

Even though nutrition transition and dietary acculturation are distinct processes with their own definitions, they have many similarities. Both are concerned with consumers’ encounter with increased availability of certain types of food that are easily favored, usually energy dense and highly processed, such as sugary drinks, snacks, and convenience foods. However, they also include a more stable supply of fresh foods, such as meat, fish, fruits, and vegetables. The process that immigrants from low-income countries (and often from rural areas), moving to high-income countries (and often to urban areas) go through, can be described as a more abrupt and radical form of nutrition transition.

The nutrition transition in the country of origin may affect the process of dietary acculturation after migration in several ways. If energy-rich and highly processed foods have become status foods, which very few can afford, it is likely that these foods also would be favored in the new country; and if more available and affordable, this would speed up the process of change. On the other hand, if the nutrition transition in the country of origin has come far, and people are using these foods already before they migrate, there might be less dietary change after migration.

## Dietary intake and changes after migration

Very few studies have compared the diet of migrants with similar ethnic groups in the country of origin. Some of the studies rely on the immigrants’ own assessments of the dietary changes they have made. Nevertheless, most studies have compared dietary intake between one or more ethnic minority groups and the majority ethnic group, or they compare the diet between different ethnic minority groups in the host country (3, 5, 19–21, 30–32). Valuable information on dietary changes after migration can also be retrieved if the results from the latter studies are compared with studies of dietary habits in the country or region of origin. In the following discussion, foods will be divided into staple, complementary, and accessory foods, as suggested by Kocturk-Runefors (29). Some of the main features of the traditional diets will be described, before the dietary habits after migration are discussed.

### Staple foods

Most of the traditional diets of migrants from low-income countries contain large amounts of carbohydrate-rich staple foods, mostly cereal grains. In India, commonly used grains are wheat, rice, sorghum, and pearl millet, with different consumption patterns of these grains in the various parts of the country (24). In an overview from India, the grain and pulse intake is shown to vary in the different states from a little over 300 to almost 600 g/day/Consumption Unit (CU) (33). The main staple in Pakistan and some parts of India is *chapatti* or *roti* made from *Atta* (whole grain wheat) flour; in Bangladesh and Sri Lanka, it is rice (34, 11).

The immigrants from low-income countries in Europe have met an abundance of highly processed foods to which accessibility may be high and nutritional knowledge low (35). A high consumption of highly processed foods, such as burgers, pizzas, French fries, chips, cakes, biscuits, and sweetened breakfast cereals among some immigrant groups have been described (34, 36). The concomitant reduction in whole grains has occurred even for those adhering to traditional staple foods, for example *chapatti*, by exchanging the traditional *Atta* flour with white flour. Simmons and Williams found that this habit varied highly between different South Asian groups in Coventry, England, from 29% among Pakistanis to 93% among the Gujaratis (30). A study among Pakistani immigrants in Norway found a relatively low grain intake (190 g/day/adult woman), similar to that of ethnic Norwegians (37), and a large part was commercial Norwegian bread, often made with a very small proportion of whole grain (38).

### Complementary foods

Many traditional diets contain generous amounts of pulses (22, 33). The consumption of fruits and vegetables may vary, and are subjected to seasonal variations (33, 35). Some South Asian regions may have very low consumption of fruit and vegetables. For example, a study from India showed that the average consumption of fruit and vegetables varied by state from 56 g/CU/day to 350 g/CU/day (33). Thus, all were below the World Health Organisation (WHO) recommendations of ≥ 400 g/person/day (39). The intake of meat, fish, and dairy products also varies from essentially vegetarian areas to more meat/fish eating areas. However, due to poverty as well as culture or religion, the intake of meat/fish and dairy is often low, even in nonvegetarian areas.

Among the often reported changes by migrants to high-income countries is a reduction in intake of pulses and an increase in intake of meat and sometimes also of fish. In a study in Oslo, Norway, 45% of Pakistani and 63% of Sri Lankan immigrants reported to have increased their meat intake, while 30% and 45%, respectively, reported to have reduced their intake of beans and lentils (31). They also increased their intake of dairy foods. A qualitative study on Pakistani women indicated that mainly high fat dairy products, such as full fat yoghurts, milk, and cheese were consumed (35). In a study from Britain, Vyas et al. (40) found that lamb and chicken contributed substantially to the energy intake among Pakistani immigrants, whereas pulses were not among the substantial (top 10) contributors to energy intake. Large variations in changes in consumption of vegetables have been described, probably reflecting both the food habits in the country/region of origin and in the new country. Among the Pakistani and Sri Lankan immigrants to Norway, many reported to have increased their consumption of vegetables after migration (31). Osler and Hansen (32) reported a higher vegetable intake among immigrant school children compared to their Danish counterparts. On the contrary, Simmons and Williams (30) reported that South Asians in Coventry ate vegetables less frequently than their European counterparts. The Sri Lankan immigrants in Norway came from areas with traditionally high vegetable consumption, but being refugees from a country in war may explain why they reported increased consumption, despite the fact that vegetables are comparatively more expensive. Pakistani immigrants have reported strong seasonal variations in fruit and vegetable intake in Pakistan (35). Thus, the reported increase may be a reflection of more stable consumption of fruits and vegetables all year around in Norway.

### Accessory foods

Most of the traditional diets are low in energy density, fat-rich foods and sugary drinks. The changes that have occurred in the immigrants place of origin due to the nutrition transition may differ according to country, urban/rural place of living, and socioeconomic standing. These factors will have a bearing on the changes after migration.

Among immigrants from Sri Lanka and Pakistan in Oslo (31), the majority reported to have increased their fat consumption after migration. For the Pakistanis, it was mostly the oil consumption that increased, whereas for the Sri Lankans it was both butter and oil. A study from Birmingham (41) showed that South Asians were more likely to deep fry and use butter, ghee, and full fat milk than the other ethnic groups (Europeans and Afro-Caribbeans).

The most reported dietary features among migrants in Europe are the high consumption of sugary snacks and drinks, and fat-rich foods and snacks, especially among the younger generation. This is in agreement with the model described by Koctürk-Runefors (29). In Denmark, Osler and Hansen (32) reported that immigrant school children aged 12–14 years consumed greater amounts of sugar from cakes than their Danish counterparts. A study from Oslo (36) showed that 15–16 year olds from South Asia consumed sweets and sugary soft drinks more often than their counterparts from minority groups originating from Eastern Europe and Western high income countries.

### Implications for changes at nutrient level

Due to the high intake of grains, legumes, and tubers, the energy percent from carbohydrates in many traditional diets is high, usually 60–70% (11, 24, 42, 43), whereas the energy percent from fat is low. The fat energy percent has been shown to vary from 13% in Tanzania (rural/urban) to 25 and 27% in Sri Lanka and India, respectively, (urban and semi urban) (11, 44). There are also differences in fat intake according to region and urban/rural settings, with a higher availability and purchasing power in the urban areas (8).

The most striking changes after migration seem to be in total energy intake and percent of energy from carbohydrates. When British Gujaratis from Sandwell, United Kingdom, were compared with their counterparts of the same age from similar cultural and genetic background in Gujarat in India, the energy intake was much higher in the UK group both for men: 2,330 kcal (9,749 kJ) vs. 1,440 kcal (6,025 kJ) per day and women: 1,690 kcal (7,071 kJ) vs. 1,210 kcal (5,063 kJ) per day (14). For men and women in Sandwell, dietary energy from fat was higher (39/40%) and was lower from carbohydrate (48/43%) compared to those living in Gujarat (fat: 31/32%, carbohydrate: 56/55%). Dietary folate was much higher in Gujaratis from Sandwell (men/women; 215/137 mg/day) compared to those living in Gujarat (81/73 mg/day), whereas no significant difference was found for vitamin B_12_.

Anderson et al. (45) compared 1st and 2nd generation South Asian women with a sample from the general population in Glasgow, Scotland. They found a higher energy intake among both Asian (7,950 kJ) and British born (7,760 KJ) South Asians, compared to the general population (6,975 KJ). The contribution of fat to energy intake was high (42.4%) for first generation South Asians, similar to the general population (39%). First generation South Asians had more energy (15%) from saturated fat, while the next generation had 13%, which was similar to the general population. South Asians had a lower intake of vitamin C (5.1 mg/100 kJ) compared to the general population (6.8 mg/100 kJ).

The relatively high fat intake among South Asian migrants to GB has also been shown in some earlier studies. In a review including nine small studies, all reported an intake of energy from fat over 35% and six showed a fat energy percent of 39 or above (46). The high energy intake among immigrants may start early. A study of 9- to 10-year-old children in the United Kingdom (47) showed that South Asian children had a higher energy and fat intake and lower carbohydrate intake than white European children and black African-Caribbean children. The intake of vitamin C and D, as well as the intake of energy from sugar, was lower than that of the other groups.

Data from a study of Pakistani women in Oslo showed that the high fat intake may be a problem also in other host countries than GB (38). Energy from total fat was 40% and from saturated fat 13%. Similar to the studies above, the intake of energy from carbohydrates was 44%, which is very close to that of the general population (37). Intakes of fiber (17 g/day), vitamin D (1.3 µg/day), folate (177 µg/day), calcium (494 mg/day), and iron (8.7 mg/day) were far below the recommendations. This study also showed that a large part of the energy (over 60%) was taken late in the day, after 17 o'clock.

One study from 2003 (40) and a few older studies (48, 49) have reported lower energy intake among South Asian immigrant groups compared to their European counterparts. Thus, the dietary picture of immigrants from low-income countries is complex. However, with a few exceptions, the general picture for South Asians after migration is a substantial increase in energy and fat intake and a reduction in carbohydrates. Concomitant with this reduction there has been a switch from whole grains to more refined carbohydrates, which has resulted in a low intake of fiber, as shown in the study from Norway (38). The proportion of energy from saturated fat and polyunsaturated fatty acids (PUFA) has varied in the different studies. It has earlier been shown that the intake of n-3 PUFA may be especially low, whereas that of n-6 PUFA is high among immigrants from South Asia (49, 50).

## Important factors for dietary intake

A few studies have looked into some of the factors and processes described in the model proposed by Satia Abouta (1) to be important for dietary changes after migration. In the study from Oslo (31), it was shown that both Pakistanis and Sri Lankans had changed their meal pattern substantially from the traditional pattern in their country of origin. They had cut down the hot meals from 3 per day to an average of about 1.5. Fat, such as oil, butter and margarine, was used to a larger degree by the youngest age group (<40 years of age) and by those having a poor command of the Norwegian language, whereas foods rich in fat were used less frequently by those who had more years of education.

A number of qualitative studies among South Asians have revealed the immense social pressure to eat and prepare heavy, fat-rich food at social gatherings (35, 51). Moreover, there has been resistance to reducing the amount of butter and whole milk in food preparation. It was felt that reducing the fat content was equivalent to depriving themselves or their families (35, 51). However, these studies have also revealed the needs and wishes for specific advice from nutritionists on how to make their diet more healthful. These needs were related both to the diet of their own food culture, and the diet in their host country. Even though the adult immigrants usually prefer their own food because of taste (52), the children get acquainted with the new foods in kindergarten, at school, and when visiting friends. In one of the studies from Oslo, Pakistani women told about their helplessness when the children requested Norwegian food which they did not know how to prepare in a healthy way (35). Thus, children's wishes are important in the dietary acculturation process. Another reason for serving dishes from the host countries’ food culture is that they are considered easy to prepare and time saving (52). The use of bread, made of mostly refined flour instead of *chapatti* made of whole grain flour among Pakistani women in Norway (38) illustrates that such a change may have nutritional consequences.

Another point which has been revealed through qualitative studies is that the immigrants tend to view most foods that do not belong to their own food culture, as belonging to the food traditions of the host country (6, 52). This includes what has been called ultra-processed foods (23) rich in refined carbohydrates, such as hamburgers, pizza, and pasta dishes. Results from studies of different immigrant groups have suggested that the informants often view the diet of the host country as healthier (6, 52). Thus, the immigrants may have fewer prejudices against extensive use of these ultra-processed foods than the host population has.

Knowledge of the special disease patterns in certain immigrant groups, such as the South Asians, combined with well-meaning nutrition information from health personnel, has made many immigrants believe that their own traditional diet is unhealthy (6, 52, 53). At the same time, food culture is so tightly linked to identity that there are aspects that they will never give up. Lawton et al. (53) have put it like this: ‘Perceptions of South Asian foods: bad for health; good for self’ when describing some Pakistani diabetic men's struggle to change their diet. There are many accounts as to why the own food culture is preferred. These often refer to the staple foods, such as chapatti and rice, which are said to be the ‘foundation’, ‘soul food’, ‘our food’, ‘the only food that gives satiation’ (6, 52–54). This is in line with the model proposed by Kocktürk-Runefors (29). Such accounts also often refer to the method of preparation, where frying often is considered a part of the traditional food culture which is difficult to change (52, 55). Even though many immigrants have changed the breakfast and lunch pattern toward that in the host country, the preferences for dinner together with the family are taken from the traditional food culture (53).

## Health implications

### Energy-dense foods

As described above, South Asians tend to consume more total energy and energy-dense foods, such as fat and fatty foods, snacks, and sweets after migration. Consumption of energy-dense foods will usually lead to a higher intake of energy compared to less energy-dense foods, since appetite regulation is to some extent dependent on bulk. This may cause weight gain and subsequent overweight and obesity (56). Energy-dense foods are defined as foods containing 225–275 kcal/100 g (57). The average energy content of products from three known fast-food chains in United Kingdom were found to average 265 kcal/100 g (56). In contrast, international recommendations set by the World Cancer Research Fund Report of 2007 (57) is to keep energy density below 125 kcal/100 g to prevent obesity.

A positive association between energy density of diet and MetS, T2D, and risk of CVD has been demonstrated for South Asians (58). Results from the European Prospective Investigation of Cancer (EPIC)-Norfolk prospective cohort study of 22,000 subjects over 12 years showed that an energy dense diet was related to the development of incident T2D (59). Noteworthy, the total dietary patterns changed drastically with the change in energy density. In the lowest quintile of energy density, there was a higher consumption of fresh vegetables and fruits, lower intakes of meat and processed meat, and lower energy from fat. The authors argue that the energy density of the diet is a simple indicator of its nutritional quality (59).

Frequent intake of fast foods was shown to be strongly associated with weight gain and insulin resistance in the CARDIA study in the United States, a 15 years follow-up of 3,031 young black and white adults at baseline. This association was stronger in the white than in the black people. Compared to the lowest frequency intake, those with the highest gained 4.5 kg extra and had a two-fold increase in insulin resistance (60).

#### Dietary fats

Fat is the most energy dense macronutrient. It is therefore important to limit fat intake. Dietary recommendations generally state that fat intake should not exceed 30–35% of total energy (7, 61, 62). As described above, many studies show a fat intake in immigrant South Asians above the recommended intake. Gupta et al. (33) found a high positive correlation of CVD mortality with intake of fat (*R*=0.67) in a national study in India. Dietary fatty acids (FA) have individual metabolic effects and may affect glucose metabolism through altering cell membrane FA composition and function, including insulin receptor binding. FA consumption is reflected in the relative availability in plasma and tissue lipids. Intervention studies have shown that insulin sensitivity improves when trans fatty acids (TFA) and saturated fatty acids (SFA) are replaced with PUFA and monounsaturated fatty acids (MUFA) (63).

Total fat intake has increased in developing countries in the wake of the Nutrition Transition, especially the intake of vegetable oils. Some of these oils contain SFA and TFA such as coconut oil, palm oils, *ghee* (semifluid clarified butter), and partially hydrogenated vegetable oils, which are also often used by the migrants (64, 65).

Misra and coworkers have reviewed the literature in regard to interventions among South Asians with n-6 and n-3 PUFAs and MUFAs. Although the effect of n-6 PUFA (linoleic acid) and MUFA showed decreased blood pressure, plasma glucose, HbA1c, tot cholesterol, low density lipoprotein (LDL)-cholesterol, the effect of n-3 PUFA supplementation on insulin sensitivity and hyperglycemia has not shown convincing results, even though a more favorable lipid profile could be demonstrated. (44)

### Fiber-rich foods

#### Whole grains

The change to more refined flours and grains after migration may reduce the nutritional quality of the diet, since the refined products contain less trace minerals, vitamins, antioxidants, phytosterols, and essential FA and fiber than whole grains.

The health benefits of whole grain consumption have been well documented (66–68). The preventive effect on weight gain and central obesity of a diet rich in whole grain products compared to refined grain products has been explained by its lower energy density and a possible lower palatability which may promote earlier satiation and thus prevent overeating (67). In addition, satiation may last longer after a meal due to prolonged gastric emptying time and slower nutrient absorption. Whole grains have also been shown to prevent insulin resistance and MetS, including diabetes, dyslipidemia, and hypertension (69). Similar effects have also been shown in a study from Iran (70). Increased intake across quartiles of whole grains was favorably associated with most of the components of MetS, including glucose homeostasis, whereas increasing intake of refined grains was associated with high serum triglyceride levels and elevated blood pressure.

#### Fruits and vegetables

For those migrants that end up eating less fruits and vegetables after migration, the risk of diabetes or MetS may increase. A meta-analysis of 13 cohort studies showed that increased consumption of fruits and vegetables from less than three to more than five servings per day was related to a 17% reduction in risk of coronary heart disease (fatal and nonfatal myocardial infarction) (71).

Esmaillzadeh et al. (72) found in a cross-sectional study of female teachers in Iran that those in the highest quintiles of both fruit and vegetable intake had 34% and 30% lower adjusted chance, respectively, of having MetS compared to those in the lowest quintile of intake. The adjusted effects of both fruits and vegetables were significant on almost all the different components of MetS and was particularly strong with regard to blood pressure. The average daily intake of fruits and vegetables was 414 g, and the highest and lowest quintiles spanned from 98 g to 362 g for fruits and 142 g to 307 g for vegetables. In comparison, a 24-h dietary recall study on Pakistani women showed that mean intake of fruits and vegetables was 355 g/day (38).

Another meta-analysis of six prospective cohort studies found that only cruciferous and green leafy vegetables had a significant association with the incidence of diabetes. An increase of 1.15 servings per day was associated with a reduction of 14% hazard ratio for T2D (73). A negative correlation of CVD mortality with green leafy vegetable intake (*r*= − 0.42) has also been found for the population of India (33).

In the EPIC cohort study with a 12-year follow-up, T2D incidence was shown to be inversely related to fruit and vegetable intake and more strongly to vitamin C level in plasma. The odds of getting diabetes was 62% lower for those in the top quintile of plasma vitamin C (74).

#### Pulses

The reduced consumption of pulses may also have relevance for T2D. Pulses are good sources of fiber, amylose starch, and phytochemicals, such as phenols, lectins, and phytates which lead to a slow absorption of their content of carbohydrates from the gut (75). In other words, they have a low glycemic index (GI). In the Shanghai Women's Health cohort study, a reduced incidence of T2D was related to an increased intake of pulses. The adjusted relative risk comparing the upper quintile to the lowest was 0.62 for total intake of legumes (76).

A literature review and meta-analysis on the glycemic effects of non-oil-seed pulses taken alone or in high fiber diets confirmed that intake of pulses improve long-term markers of glycemic control in humans, such as fasting blood glucose, glycated blood proteins (HbA_1C_), and insulin resistance (75).

#### Fiber-rich foods taken together

The sources of fiber as described above will, taken together, have a positive effect both on glycemic control and levels of serum lipids, as well as lower blood pressure and have anti-inflammatory properties. The NIH-AARP Diet and Health Study on a 9-year prospective cohort investigating more than 30,000 deaths demonstrated that dietary fiber was inversely associated with risk of total death and death from CVD, infectious diseases, and respiratory diseases. Fiber from grains showed the most consistent inverse relation with risk of total death and cause specific death and reduced the risk of CVD deaths by 24% (77).

Earlier research from India encompassing short-term and long-term dietary intervention on diabetic patients, using a high carbohydrate high fiber (HCHF) diet containing 67% carbohydrate and 14% fat, demonstrated improvement in the postprandial control of blood glucose and a lowering of serum insulin and serum lipids (78). This diet contained 52 g fiber, which is more than the 20–35 g usually recommended and much more than the intake of the average American, which is 14 g (79). Visnawathan et al. (78) argues that the HCHF diet is similar to the Indian traditional dietary pattern and therefore easy to adhere to, also in the long term.

Gaesser concluded after reviewing the literature that a high-carbohydrate low-fat diet with emphasis on fiber-rich carbohydrates, particularly cereal fiber, will have beneficial influence on health and weight control (80).

### Refined carbohydrates including sugar

#### Refined carbohydrates

Refined grains and refined starch from other sources, as well as simple sugars, have high GI (defined as the incremental area under the glucose response curve after an intake of a standard amount of carbohydrate in the test food relative to the same amount of a reference food – usually white bread or glucose) (81). A diet with a high glycemic load (amount of carbohydrate multiplied by GI) has been associated with high postprandial blood glucose and risk of T2D as well as high plasma triglycerides (TG) and low high density lipoprotein (HDL) levels (82). However, the effect of postprandial glucose excursions on glucose homeostasis in the long term is still debated (83)

The association between refined cereal consumption and MetS has been investigated in India in the Chennai Urban Rural Epidemiology Study (84). About half the daily energy intake came from refined grains, including white polished rice and products made from refined wheat flour and semolina. Rice, the major staple, contributed three-fourths of the mean intake of 253 g/day. Comparing quartiles of energy adjusted intakes of refined grains, the odds ratio for MetS in a multivariate model was 7.8 between the highest and the lowest quartiles. The intake was associated with all the components of MetS.

The glucose response to an oral glucose tolerance test has been shown to vary according to ethnic group with the same BMI. Indians have been shown to have a higher prevalence of glucose intolerance than many other ethnic groups, and the risk starts to increase at very low levels of BMI (85). It is proposed that high risk ethnic groups in regard to developing T2D are ‘high responders’ to glycemic loads (86) and thus may have an increased risk if exposed to more highly processed sources of carbohydrates.

White rice has a fairly high GI, varying from 56 to 72 depending on type of rice. An association between white rice intake and risk of diabetes has been found in recent studies (69, 87, 88). A meta-analysis of seven prospective cohort studies, including both Asian (Japanese and Chinese) and Western populations, concluded that the pooled relative risk of diabetes for the Asians, comparing the highest intake of rice with the lowest, was 1.55 (87). Similarly, Shi et al. (88) found in a 5-year follow-up study of adults in China a strong positive association between rice intake and fasting hyperglycemia. However, the rice intake was inversely related to weight gain and the risk of hypertension. Consequently, there was no association with MetS. Rice intake in the highest tertile compared to the lowest yielded an adjusted odds ratio of 2.5 for fasting hyperglycemia. The consumption of rice in this population was however very high, the mean intake being 321 g/day, much higher than that found in rice eating immigrant minorities in Europe who consume about 200 g cereals in total.

Individuals being diabetes prone, who consume rice on a daily basis, may thus experience postprandial glycemia, insulinemia over a prolonged period throughout the day, especially if the meal is heavy. Burden et al. (89) showed in a cross-over experiment in United Kingdom on 15 healthy multiethnic volunteers given a typical Asian and a European meal that a higher degree of glycemia and insulinemia was experienced after consuming the Asian meal. The Asian meal contained white rice as main carbohydrate, but the two meals were isocaloric. Fasting glucose and insulin levels were not achieved before 6 h after the Asian meal, whereas they were normalized 2 h after the European meal. It could be that the intake of rice in the case of the European minorities, in whom the consumption usually is concentrated to one daily meal, could prolong the glycemic response to this meal. Misra et al. (90) have suggested, in regard to South Asians, to spread the intake of carbohydrate more evenly throughout the day, instead of concentrating it to one heavy meal in the evening.

#### Sugar-sweetened beverages

Increased intake of sugar-sweetened soft drinks among South Asian immigrants may amplify risk of T2D by promoting weight gain and by increasing blood glucose and insulin (91, 92). It seems that intake of extra energy ingested as sugary liquids is not fully compensated by a reduction in energy intake from a concurrent or a subsequent meal and would thus induce weight gain. Intake of sugary drinks may lead to rapid increase of blood glucose and insulin, which with frequent consumption can lead to glucose intolerance and insulin resistance, inflammation, and eventually β-cell dysfunction (91, 92). High intake of fructose (both from sucrose and from corn syrup in soft drinks) may also increase the risk of MetS through elevated hepatic *de novo* lipogenesis causing dyslipidemia as well as hypertension and accumulation of visceral fat (91, 92).

### Meat and dairy products

#### Meat

The increased intake of meat which has been described after migration may also have an impact on diabetes risk. An association between intake of red or processed meat and T2D was found in a 14-year follow-up of the Nurses’ Health Study of 69,554 women in the United States (93). This relationship has been further examined in a meta-analysis of 12 cohort-studies by Aune et al. (94). They found an increase in relative risk of diabetes amounting to 21% for red meat and 41% for processed meat when comparing high and low intake of these products. In the 26 years of follow-up of the Nurses’ Health Study (95), meat intake was also related to risk of coronary heart disease. Intake of red meat products (including processed meat) was associated with risk of myocardial infarction, nonfatal and fatal, whereas intake of fish and poultry were associated with reduced risk. A reduction in risk of coronary heart disease was found in this study when one daily serving of meat was substituted with one serving of: low fat dairy (13%), poultry (19%), fish (24%), nuts (30%), or beans (34%).

A cross-sectional study on 150 South Asian Indians living in the United States found a 70% increase in the odds of diabetes for every standard deviation in grams of protein intake per day. The difference in effect between animal and vegetable protein could not be determined due to small sample size (96). However, Fung et al. (97) found in the nurses’ Health Study that animal sources of protein were associated with a higher cardiovascular mortality than vegetable sources of protein.

#### Dairy products

The relation between intake of dairy products and T2D or MetS has been investigated by Azadbakht et al. (98) on 827 adults (18–74 years) living in Tehran. They found an inverse association between intake of dairy foods and MetS, including all the components of MetS except blood glucose (fasting) and serum TG. However, the intake of fruits, vegetables, and grains increased with a higher intake of dairy, while the intake of meat decreased and the intake of fat stayed level with increasing dairy intake. Even so, the protective effect persisted after a multivariate logistic regression that included all the above mentioned food groups, as well as level of physical activity and smoking. Differential effects of level of fat content in dairy products on risk of coronary heart disease were found by Bernstein et al. (95); increased intake of high-fat dairy showing a significant independent association with myocardial infarction.

### Conclusion and implications for prevention

To conclude, South Asians living in Europe tend to have changed their diet after migration toward a unhealthier pattern leading to a higher risk of obesity, T2D, and cardiovascular disease. The changes that have occurred follow to some extent the trajectories described in the model suggested by Kocktürk-Runefors (29), whereby ‘accessory foods’ are the first foods to be exchanged with host country foods leading to a higher intake of fatty and energy-rich foods. In regard to ‘staple foods’, a decrease in intake is likely to occur and a partly substitution of whole grains with refined sources of carbohydrate. The intake of the ‘complementary foods’, such as meat and dairy, are usually increased, whereas fruit and vegetables often are consumed to a lesser extent. In sum, these changes result in higher intake of energy relative to need, less fiber, and lower intake of micronutrients and antioxidants found in plant-based foods.

A first priority in diabetes prevention for high-risk minority populations must be to prevent further increase in weight, or if already glucose intolerant, to promote weight loss through diet modification. Important measures to prevent obesity and obesity-related diseases are to avoid energy-dense foods, ultra-processed foods, and sugar-sweetened soft drinks and nectars. Adoption of a low-energy density – high fiber diet could be the most realistic way of maintaining or losing weight in the long term, since such diet is more favorable in terms of quality. Whole grains or products derived from them, as well as fruits, vegetables, and pulses would all have health-promoting effects because of higher content of micro-nutrients, phytochemicals, and fiber. Such diet is less energy-dense due to larger bulk. Furthermore, it could also represent a more culturally acceptable diet for most migrants, if used and cooked according to traditional food habits, since it would be more likely that they originate from areas where high carbohydrate, high fiber foods were the staples. Besides, such a dietary regimen would have a greater chance of succeeding since restrictions on amounts of intake would be less important because of bulky content implying that satiety will be reached and last for a longer period after a meal. Energy intake on such a diet may thus decrease spontaneously without having to go through the effort of slimming.

The diet should contain all the three food groups that are the main contributors to fiber: whole grains, fruits and vegetables, and pulses. For rice-eating minority groups, it should be recognized that it could be difficult to switch from white rice to whole grain. However, since many of them have replaced traditional rice-containing meals with more European versions, encompassing breakfast cereals and bread, it would be more pertinent to recommend whole grain varieties of these products. Green leafy and cruciferous vegetables have been found to have a particularly beneficial effect on diabetes risk and should be included in the recommendation of vegetable intake.

Many minority populations in Europe are genetically prone to developing diabetes and are ‘high responders’ to carbohydrate in terms of postprandial glycemic response. Avoiding long-lasting postprandial glucose excursions may be important in subjects that are high responders, since this could contribute to insulin resistance, glycation of plasma proteins, oxidative stress, and inflammation (99). It would therefore be advisable to apply the concept of GI when planning dietary composition for these ethnic groups. Even if the clinical significance of postprandial glucose excursion is still debated among scientists, it would surely not add to the risk of developing glucose intolerance if this principle is adhered to.

Most European dietary recommendations for dietary fat are set to a range of 20–35% of energy intake. The quality of the fat is important since it may affect insulin sensitivity and have a direct influence on the insulin receptor of the cell. Recommendations for intake of saturated fat are set to below 10%. Diets with a high proportion of industrially processed foods, including biscuits, cured meats, and fatty snacks, will often exceed this recommendation. In addition, red meat and especially processed meats that contain SFA should be avoided, likewise high-fat dairy products. Riserus et al. (63) contend that improving fat quality should be part of a dietary lifestyle strategy to prevent or manage T2D. They suggest replacing fats from red meat and butter with nonhydrogenated oils and margarines rich in MUFA and PUFA to improve insulin sensitivity and reduce serum LDL/HDL ratio and TG, thus reducing risk of diabetes and CVD. They also point to nuts and seeds as being excellent sources of both MUFA and PUFA.

Meal pattern and the total energy intake at each meal should also be taken into consideration. A large meal containing both a high carbohydrate load and a high proportion of fat may lead to a high and long-lasting blood glucose level after the meal. In this case, excess glucose will most likely be part of *de novo* synthesis of fat in the liver and lead to an increase formation of very low density lipoprotein (VLDL), which is converted to LDL, the main atherogenic lipoprotein. For some ethnic groups, this is of high significance, especially for those who tend to have the biggest meal at the end of the day before going to bed. This could make the effect of high blood glucose and TG even more deleterious since physical activity after the meal would most likely be extremely low. Lunde et al. (100) have showed that even low physical activity after a meal (slow walking) attenuated the blood glucose response considerably in high-risk Pakistani women.

The process of dietary change described in this review may have started already before migration, due to the nutrition transition also occurring in the immigrants country of origin, The pattern and speed of change in diet may differ from country to country. Thus, the extent and nature of change after migration can be expected to vary according to place of origin and time of migration. As suggested in the model of dietary acculturation proposed by Satia-Abouta et al. (1), the dietary changes are governed by sociodemographic, economic, and cultural factors as well as the extent of exposure to host country. This implies that immigrants, even those belonging to the same ethnic group, may be at very different levels in the acculturation process. This may explain some of the conflicting results in the studies presented here. It also implies that efforts to prevent nutrition related diseases in these population groups must be planned with these differences in mind to be effective.
